# Diagnostic Performance Evaluation of the GXT96 X3 Extraction System with the FluoroType^®^ SARS-CoV-2 varID Q Assay for SARS-CoV-2 Detection and Mutation Screening

**DOI:** 10.3390/diagnostics16131951

**Published:** 2026-06-23

**Authors:** Riffat Munir, Oluwakemi Laguda-Akingba, Lesley Erica Scott, Wendy Susan Stevens

**Affiliations:** 1 Wits Diagnostic Innovation Hub, Faculty of Health Sciences, University of Witwatersrand, Johannesburg 2193, South Africa; lesley.scott@wits.ac.za (L.E.S.); wendy.stevens@wits.ac.za (W.S.S.); 2 Department of Laboratory Medicine and Pathology, Faculty of Health Sciences, Walter Sisulu University, Mthatha 5117, South Africa; 3 National Health Laboratory Service, Gqeberha 6001, South Africa; 4 National Priority Program, National Health Laboratory Services, Johannesburg 2131, South Africa

**Keywords:** SARS-CoV-2, molecular diagnostics, RT-PCR detection, variant mutation, diagnostic performance evaluation

## Abstract

**Background**: The continued evolution of severe acute respiratory syndrome coronavirus 2 (SARS-CoV-2) created ongoing challenges for molecular diagnostics and variant surveillance. Assays capable of maintaining diagnostic sensitivity across emerging variants while providing variant-related information remain essential for clinical and public health applications. This study evaluated the performance of the GXT96 X3 extraction kit in combination with the FluoroType^®^ SARS-CoV-2 varID Q version 1.0 assay (Hain LifeScience SA (Pty) Ltd., South Africa) for the detection, semi-quantitative assessment, and variant characterization of SARS-CoV-2 under laboratory conditions. **Methods:** A total of 220 samples were evaluated, including residual nasopharyngeal clinical specimens (*n* = 183), reference materials, and cultured SARS-CoV-2 virus dilutions. Residual specimens collected during multiple COVID-19 waves in South Africa (wild-type, Beta, Delta, and Omicron) were compared against standard-of-care (SOC) molecular assays used for routine diagnosis. RNA extraction was performed using the automated GXT96 X3 platform, followed by amplification on the FluoroCycler^®^ XT using the FluoroType^®^ SARS-CoV-2 varID Q assay targeting *RdRp* and *N* genes, with additional spike gene mutation detection for variant detection. Diagnostic accuracy, agreement (Cohen’s kappa), precision, linearity, and limit of detection (LoD) were assessed. **Results:** The assay demonstrated a sensitivity of 98.4% (95% CI: 94.2–99.8) and specificity of 100% (95% CI: 95.9–100.0) compared with SOC assays, with an overall agreement of κ = 0.981. Precision analysis showed acceptable reproducibility with a standard deviation of ≤1.49 and a coefficient of variation of ≤3.83%. Regression analysis demonstrated linearity across the dilution series (R^2^ = 0.9882 for *RdRp* and 0.994 for *N* genes). The LoD was ≤100 copies/mL for the *RdRp* gene and 250 copies/mL for the *N* gene. Variant-associated spike mutations corresponded broadly with epidemiological wave patterns observed in South Africa. **Conclusions:** Under the evaluated laboratory conditions, the GXT96 X3 extraction platform combined with the FluoroType^®^ SARS-CoV-2 varID Q assay demonstrated high diagnostic accuracy and reproducibility for SARS-CoV-2 detection across a range of viral loads with additional spike gene mutation detection as an adjunct feature.

## 1. Introduction

Severe acute respiratory syndrome coronavirus 2 (SARS-CoV-2), the causative agent of Coronavirus disease 2019 (COVID-19), emerged in late 2019 and rapidly evolved into a global pandemic. SARS-CoV-2 is an enveloped, single-stranded, positive-sense RNA virus that is transmitted between humans, predominantly via respiratory droplets and aerosols [1,2,3,4]. During the pandemic, SARS-CoV-2 exhibited substantial genetic diversity driven by ongoing viral replication and selective pressure, resulting in the continual emergence of novel variants with altered transmissibility, virulence, and immune escape properties. To support global surveillance and risk assessment, the World Health Organization (WHO) classified SARS-CoV-2 variants into variants of concern (VOCs) or variants of interest (VOIs) based on their epidemiological and biological characteristics [5,6,7].

Successive pandemic waves were dominated by the Alpha, Beta, Gamma, Delta, and Omicron VOCs [8], each demonstrating increased transmissibility compared with the ancestral Wuhan strain [7,8,9]. The Omicron variant, first identified in southern Africa in November 2021 [10], rapidly became the globally dominant lineage and subsequently diversified into numerous sub-lineages. This extensive diversification posed challenges for diagnostic accuracy and highlighted the need for robust molecular detection methods capable of maintaining sensitivity across evolving variants, as well as for scalable and timely variant surveillance strategies [11].

Nucleic acid amplification tests (NAATs) performed on upper or lower respiratory tract specimens remain the reference standard for the laboratory diagnosis of SARS-CoV-2 infection [2]. These tests include real-time reverse transcription polymerase chain reaction (RT-PCR), loop-mediated isothermal amplification (RT-LAMP), and sequencing-based approaches [12,13,14]. Most molecular assays target conserved regions of the SARS-CoV-2 genome, including the *E*, *S*, *N*, *RdRp*, and *ORF1ab* genes [4,13], to minimize the impact of viral genetic variation on assay performance. With regard to viral load estimation, cycle threshold (Ct) values are commonly used as a proxy for viral load; however, they are highly assay and platform-dependent, limiting comparability and clinical interpretability. Despite these limitations, viral load has been associated with disease severity, transmission risk, and clinical outcomes, particularly among immunocompromised and other high-risk populations [15,16].

Whole-genome sequencing (WGS) is the gold standard for comprehensive SARS-CoV-2 variant characterization and surveillance; however, its widespread implementation is limited by cost, turnaround time, and the requirement for specialized infrastructure and technical expertise [4]. Maintaining diagnostic readiness remains essential, necessitating continued development and performance monitoring of molecular assays capable of reliable SARS-CoV-2 detection and concurrent mutation-based identification of clinically relevant variants to inform public health responses and clinical decision-making.

This laboratory-based study aimed to evaluate the diagnostic performance of the GXT96 X3 extraction kit in combination with the Fluorotype^®^ SARS-CoV-2 varID Q version 1.0 assay (Hain LifeScience SA (Pty) Ltd., Centurion, South Africa) for the detection, semi-quantitative assessment, and characterization of SARS-CoV-2 nucleic acid in residual nasopharyngeal and oropharyngeal specimens. The assay’s ability to detect a limited set of spike mutations was additionally assessed against epidemiological wave data. The findings provide laboratory-specific performance data to support sustained diagnostic accuracy in the context of viral evolution and contribute to laboratory preparedness for future respiratory pandemics.

## 2. Materials and Methods

### 2.1. Study Setting

This study was conducted at the National Health Laboratory Service Main Branch Laboratory, Gqeberha, Eastern Cape, South Africa. Ethics approval was granted by the University of the Witwatersrand Human Research Ethics Committee (reference number M1911201) to access residual clinical specimens following routine testing conducted for patient management.

### 2.2. Study Design

This study was designed as a laboratory-based performance evaluation using available residual clinical specimens, reference materials, and viral culture dilutions. The clinical specimen size was primarily determined by specimen availability across circulating variant waves and by the need to include a broad range of viral loads and specimen types for comparative performance assessment. Nevertheless, to ensure statistical adequacy, the minimum required sample size was calculated using Buderer’s formula [17] for diagnostic test studies, assuming an expected sensitivity of 95%, a 95% confidence level, a precision of 5%, and an estimated disease prevalence of 50%. This calculation yielded a minimum target of approximately 146 specimens. The final number of residual specimens included in the study (*n* = 183) exceeded this threshold.

### 2.3. Residual Clinical Specimens

Residual clinical oropharyngeal or nasophryngeal swabs (*n* = 183), preserved at −80 °C in phosphate-buffered saline (PBS) (Gibco, Life Technologies, Bleiswijk, The Netherlands), universal transport medium (UTM), or viral transport medium (VTM) were collected during the four COVID-19 waves across South Africa, namely wave 1 (wild-type strain), wave 2 and early wave 3 (Beta variants), wave 3 peak (Delta variants) and wave 4 (Omicron variants). These specimens were obtained from patients visiting healthcare centers for routine SARS-CoV-2 testing, and were tested on the standard-of-care (SOC) assay available at the time immediately upon arrival at a testing facility (Cobas^®^ SARS-CoV-2 (Roche Molecular, Pleasanton, CA, USA), Xpert^®^ Xpress SARS-CoV-2 (Cepheid, CA, USA), Alinity m SARS-CoV-2 AMP Kit (Abbott Laboratories, Chicago, IL, USA), and TaqPath™ COVID-19 CE-IVD RT-PCR Kit (Thermofisher Scientific, Waltham, MA USA)). Supplementary Table S1 summarizes the results obtained on these platforms. The specimens were retrieved from −80 °C storage and thawed. RNA was extracted as described below on the automated GXT 96 X3 (Hain LifeScience SA (Pty) Ltd., Centurion, South Africa). Positive specimens were selected across a range of viral loads (VLs) based on the SOC comparator method Ct values used for initial SOC testing (*E* gene range: 14.90–37; *N* gene range: 10.90–34.80; *S* gene range: 10.60–33.30; *ORF1ab* gene range: 10.0–35.40).

### 2.4. Reference Material

Accuplex wild-type SARS-CoV-2 whole-genome reference material (SeraCare, Milford, MA, USA) consisting of an undiluted sample (5000 copies/mL) was tested in singlicate. Thereafter, four dilutions (1000 copies/mL, 500 copies/mL, 250 copies/mL and 100 copies/mL) were each tested in triplicate (*n* = 13). A negative Accuplex reference sample was also included (*n* = 1). In addition, known Accuplex reference of UK, SA and Brazil variants (all of SeraCare, Milford, MA, USA) at a concentration of 1000 copies/mL were each tested in duplicate (*n* = 6). RNA extraction was carried out on the automated GXT 96 X3 platform as described below.

### 2.5. SARS-CoV-2 Viral Culture Samples

SARS-CoV-2 viral cultures were obtained through collaborations with Professor Wolfgang Preiser from the Department of Medical Virology, Stellenbosch University, South Africa, and Professor Bavesh Kana from CBTBR, University of the Witwatersrand, South Africa. The original viral culture concentration was calculated using semiquantitative PCR in the laboratory, yielding an estimate of 1 × 10^5^ copies/μL. In a biosafety level 3 laboratory, four serial dilutions of the viral supernatant in PBS were prepared: 1:1000, 1:10,000, 1:100,000, and 1:1,000,000 (approximately log 5.0, log 4.4, log 3.4, and log 2.5 viral copies per milliliter [cp/mL]). These were prepared in 15 mL Falcon tubes (Thermo Fisher Scientific, Waltham, MA, USA). Nest Biotechnology oropharyngeal specimen collection swabs (Whitehead Scientific Pty., Ltd., Modderfontein, Lethabong, South Africa) were used to prepare swabs of the different dilutions via a swab capture technique. Each swab was immersed and swirled in the designated dilution for 30 s (15 s clockwise, 15 s counterclockwise). Swabs were then placed into labeled cryovials and sealed. Each swab was resuspended in 1 mL of PBS, vortexed vigorously for 1 min, and left at room temperature for 10 min before testing on a standard care assay and further evaluation. Each dilution was tested in triplicate (*n* = 12).

### 2.6. FluoroType^®^ SARS-CoV-2 varID Q Assay

The FluoroType^®^ SARS-CoV-2 varID Q Ver 1.0 kit (Hain LifeScience SA (Pty) Ltd., Centurion, South Africa) is an in vitro, quantitative test for the detection of two independent SARS-CoV-2 viral RNA targets (*RdRP* and/or *N* genes) while simultaneously differentiating SARS-CoV-2 variants from the wild-type virus by detecting mutations in the spike gene which include del69-70, N501Y, D80A and E484K. The kit includes Amplification Mix A (AM-A), Amplification Mix B (AM-B), low and high positive standard set and positive control DNA (C+). The assay makes use of a separate Universal Internal Control 2 Ver 1.0 kit (U-IC2), which is an encapsulated polynucleotide RNA Internal Control (IC), mimics lysis of viral particles and serves as an extraction, reverse transcription and amplification control. The kit uses real-time PCR technology to amplify RNA from upper respiratory specimens such as nasopharyngeal swabs and lower respiratory specimens such as bronchoalveolar lavages and tracheal aspirates.

### 2.7. Nucleic Acid Extraction

Nucleic acid extraction on residual clinical specimens, Accuplex reference material and live viral culture dilutions was performed according to the manufacturer’s instructions, using the GXT96 X3 extraction kit (Hain LifeScience Pty Ltd., South Africa) on the GXT96 X3 automated extraction platform. Briefly, a total of 800 µL of each sample was extracted and eluted in 50 µL of elution buffer. An extraction negative control was included in each run to monitor for carry-over contamination during the extraction process, using nuclease-free water as the template.

### 2.8. Nucleic Acid Amplification

Amplification of the extracted RNA was performed using the FluoroType^®^ SARS-CoV-2 varID Q Ver 1.0 kit on the real-time FluoroCycler ^®^ XT real-time PCR machine (both from Hain LifeScience Pty Ltd., South Africa). PCR was performed according to the manufacturer’s instructions. Briefly, the PCR reaction was prepared by combining 8 µL of mastermix (3 µL AM-A and 5 µL AM-B) and 12 µL of extracted RNA sample to give a total volume of 20 µL. Samples of negative and positive controls were put in the respective wells of a 96-well plate. For the positive control, 12 µL of C+ was used. The plate was sealed and centrifuged at 3000× *g* for 30 s. The plate was then loaded onto the FluoroCycler for real-time PCR amplification. The amplification results were analyzed manually according to the manufacturer’s instructions. The FluoroType^®^ SARS-CoV-2 varID Q Ver 1.0 assay comes with positive and negative controls to assess extraction efficiency and high and low positive controls were tested in triplicate to determine the viral loads.

### 2.9. Statistical Analysis

The results obtained on the FluoroType SARS-CoV-2 assay were compared to those obtained on the SOC primary comparator assays listed above. The statistical analysis, including the accuracy (sensitivity and specificity) and percentage agreement analyses (Cohen’s kappa coefficient), was carried out using Stata version 14 (StataCorp, College Station, TX, USA). Formal probit regression analysis was not performed due to the limited number of dilution replicates included in this evaluation study.

## 3. Results

### 3.1. Overall Results

As per the manufacturer’s instructions, results are classified as positive if the *RdRP* and/or *N* genes have a CT value of ≤40. Agreement data as well as Ct values for each gene target are provided in Supplementary Table S1. Of the 183 residual specimens tested, 5 specimens produced invalid results and were excluded from the analysis (*n* = 183 − 5 = 178). One of the positive specimens with a high Ct of 30 and 33 on *ORF1* and *E* genes, respectively, tested previously on the Roche instrument (Roche Diagnostics, Midrand, South Africa) yielded a negative result on the real-time Fluorocycler.

All the positive Accuplex reference material tested positive, and no COVID nucleic acid was detected in the negative reference material. Of the live culture dilutions, one of the 1:1,000,000 (log 2.5) culture dilutions was negative.

### 3.2. Accuracy and Agreement (Cohen’s Kappa Coefficient)

Accuracy of the GXT and the FluoroType^®^ SARS-CoV-2 varID Q Ver 1.0 assay was determined using results obtained from a total of 220 samples/tests, comprising residual clinical specimens (*n* = 178/183), Accuplex wild-type positive and negative reference material dilutions (*n* = 14), variant reference material representing country variants (*n* = 6), viral culture dilutions (*n* = 12) and assay positive and negative controls run 5 times each (*n* = 10). An overall error rate of 2.0% (5/225) was observed.

Table 1 provides the overall accuracy and agreement data. The fluorotype assay showed a specificity of 100% and a sensitivity of ≥90% compared to the SOC assays. An almost perfect overall agreement with a Cohen kappa coefficient of ≥0.9 (95% CI) was observed.

### 3.3. Precision Analysis (Reproducibility) and Linearity

**Figure 1:**
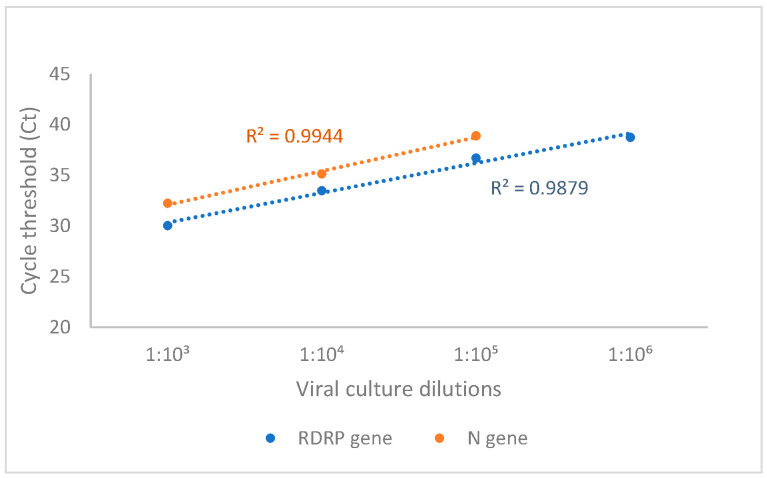
Linear regression of mean Ct (triplicate) of viral culture lysates tested across a range of dilutions (log 5–log 2.5 copies/mL) for the *RdRP* and *N* gene targets using the FluoroType^®^ SARS-CoV-2 plus Ver 1.0 assay from RNA extracted using the automated GenoXtract^®^ 96 instrument and GXT96 X3 extraction kit. Dotted lines are lines of best fit for the data points of the respective gene target. Equations of the lines are represented in the graph (R^2^: *RdRP* = 0.9879 and *N* = 0.9944).

Assay precision (standard deviation (SD) and percentage coefficient of variation (%CV)) and linearity were calculated using the reported Ct values of viral culture dilutions that were tested in triplicate (Table 2). For the *RdRp* gene, 2/3 replicates were detected at 1:1,000,000 (log 2.5) culture dilution, and for the *N* gene, no replicates were detected at this concentration. Mean Ct for the *N* gene, therefore, could not be calculated at 1:1,000,000 dilution. The FluoroType^®^ SARS-CoV-2 varID Q Ver 1.0 showed an acceptable precision with an SD ≤ 1.49 and CV% ≤ 3.83%. Regression analysis (Figure 1) showed a good linearity with R^2^ = 0.9879 and 0.9944 for the *RdRP* and *N* genes, respectively.

### 3.4. Limit of Detection (LOD)

The LoD was investigated using the Accuplex reference control material. Under the evaluated study conditions, the FluoroType^®^ SARS-CoV-2 varID Q Ver 1.0 assay was able to detect the SeraCare Accuplex reference material (for both the *RdRP* and *N* genes) at 5000, 1000, 500, and 250 copies/mL and with variability at 100 copies/mL dilution (Table 3). One replicate at 100 copies/mL dilution tested negative on the *N* gene. Replicate detection was observed down to 100 copies/mL for the *RdRp* target and 250 copies/mL for the *N* gene target. Based on the assay interpretation criteria (detection of either target), the overall assay achieved 100% detection down to 100 copies/mL.

### 3.5. Variant Detection

Of the 13 specimens collected during the Delta wave, 7 were detected without any mutation (MUT-), and 1 was positive for mutations consistent with the Beta variant. For 5 specimens, the mutation status of the locus could not be determined. Since the FluoroType^®^ SARS-CoV-2 varID Q Ver 1.0 kit is unable to detect mutations from the Delta variant, the specimens with undetected mutations could have been from the Delta wave. For the 11 specimens collected during wave 1 (WT), 8 were mutation negative (MUT-), 2 had mutations consistent with the Beta variant, and 1 came up as negative. In the case of 14 Beta wave specimens, 12 had mutations consistent with Beta variants, one was mutation negative, and one had unknown mutation status. Of the remaining presumptive Omicron wave specimens, 10 had del 69/70 mutation consistent with Alpha and Omicron variants. Gene mutation targets for SARS-CoV-2 variants are provided in Table 4.

## 4. Discussion

This study evaluated the diagnostic performance of the GXT96 X3 extraction kit combined with the FluoroType^®^ SARS-CoV-2 varID Q Ver 1.0 assay for the detection, semi-quantitative assessment, and mutation screening of SARS-CoV-2 in clinical specimens under defined laboratory conditions. Our findings show that the assay system exhibits a sensitivity of 98.4%, specificity of 100%, and an agreement score (Cohen’s kappa coefficient) of 0.981 when compared to standard-of-care molecular assays used in South African diagnostic laboratories during the study period.

The observed sensitivity and specificity meet the minimum performance criteria established by the South African Health Products Regulatory Authority (SAHPRA) for molecular COVID-19 RT-PCR kits, which require sensitivity ≥95% and specificity ≥98% [18]. These performance characteristics are comparable to those reported for other commercially available SARS-CoV-2 molecular assays. For instance, the Cobas^®^ SARS-CoV-2 test demonstrated 100% sensitivity and 97.9% specificity in a clinical evaluation [19], while the Alinity m SARS-CoV-2 assay showed 100% positive percent agreement and 96.9% negative percent agreement [20]. The agreement (κ = 0.981) observed in our study indicates that the combination of GXT96 X3 extraction and FluoroType^®^ varID Q assay performed comparably to the comparator assays under the tested conditions.

For precision analysis, the assay demonstrated a standard deviation of ≤1.49 and a coefficient of variation of ≤3.83% across the dilution series for both gene targets. These values are within acceptable limits for molecular diagnostic assays, where CV% below 5% is often considered indicative of good reproducibility [21,22]. Linear regression analysis showed linearity with R^2^ values of 0.9882 and 0.994 for the *RdRP and N* genes, respectively, suggesting the assay’s capacity for semi-quantitative viral load estimation across a dynamic range of approximately 2.5 to 5.0 log copies/mL.

The differential LoD observed between the *RdRP* and *N* genes in this study (100 vs. 250 copies/mL) suggests that the *RdRP* target may be more analytically sensitive at very low viral loads. This was further evidenced by the detection of *RdRP* in 2/3 replicates at the 1:1,000,000 viral culture dilution, whereas the *N* gene was not detected at this dilution. The inclusion of two independent gene targets (*RdRP* and *N*) in the assay design means that failure to detect one target at very low concentrations does not compromise overall positivity, consistent with international recommendations for molecular assay design [14].

The FluoroType^®^ SARS-CoV-2 varID Q Ver 1.0 assay includes detection of four spike gene mutations (del69-70, N501Y, D80A, and E484K), allowing variant mutation screening. Our analysis of residual specimens collected across successive COVID-19 waves in South Africa showed that detected mutation patterns broadly aligned with expected VOC distributions for waves where the mutation panel was relevant (e.g., Beta-associated mutations in wave 2/early 3). However, several important limitations must be emphasized. First, the assay cannot screen for Delta variants, which dominated wave 3 in South Africa, because the required mutations are not included in the panel [23,24]. In our Delta-wave specimens, 7/13 were mutation-negative and 5/13 had undetermined status, consistent with this limitation. Second, mutation profiles are not unique to specific variants; for example, the del69-70 deletion appears in both Alpha and Omicron variants [10,25]. Third, we did not perform confirmatory whole-genome sequencing; therefore, variant assignments are presumptive based on epidemiological timing.

The inclusion of viral culture dilutions and reference materials in our study design allowed for robust analytical characterization under controlled conditions. The use of the swab capture technique for preparing culture dilutions simulated clinical specimen collection more directly than spiking liquid samples, potentially providing a more realistic assessment of assay performance with clinical specimens. The invalid result rate of 2.0% (5/225) was within acceptable limits for molecular diagnostic workflows. The single false-negative result among positive specimens (a sample with Ct values of 30 and 33 on the Roche platform) likely reflects viral RNA degradation during storage or viral concentrations near the assay’s LoD rather than a systematic assay deficiency [14].

This study has several limitations. First, the use of residual frozen specimens, while practical, may have resulted in some RNA degradation and could underestimate the assay’s performance relative to fresh specimens. Second, the standard-of-care assays used as comparators varied across specimens, reflecting the real-world changes in diagnostic platforms during the pandemic but introducing heterogeneity in the reference standard. Third, the sample size for certain variant categories was limited, precluding robust statistical comparisons of variant-specific sensitivity. Fourth, the LoD assessment did not employ formal probit regression or CLSI EP17 statistical approaches; therefore, the reported LoD values should be interpreted as approximate detection thresholds under the specific study conditions. Fifth, the variant mutation screening capability was assessed against epidemiological wave data rather than whole-genome sequencing, which remains the gold standard for variant confirmation [4,12]. Future studies should include direct comparison with sequencing data to validate the accuracy of mutation detection.

The continued evolution of SARS-CoV-2 highlights the importance of ongoing diagnostic evaluation [7,8]. Variant mutation screening assays such as the FluoroType^®^ SARS-CoV-2 varID Q system may provide supplementary mutation information alongside routine SARS-CoV-2 detection. However, interpretation of these mutations is constrained by the predefined mutation panel and absence of sequencing confirmation. As such, mutation screening from this assay should be considered complementary to, and not a substitute for, sequencing-based surveillance approaches.

In conclusion, under the evaluated laboratory conditions, the GXT96 X3 extraction kit combined with the FluoroType^®^ SARS-CoV-2 varID Q Ver 1.0 assay showed diagnostic performance for SARS-CoV-2 detection comparable to the comparator methods. The assay includes limited mutation screening as an adjunct feature; however, comprehensive variant characterization requires whole-genome sequencing.

## Figures and Tables

**Table 1:** Accuracy (sensitivity, specificity) and agreement (Cohen’s kappa coefficient) of the FluoroType SARS-CoV-2 varID Q Ver 1.0 assay.

Sample Type	*n*	Sensitivity (CI)/Specificity (CI)	PPV/NPV (CI)	Cohen Kappa (CI)
As described	220	98.4% (94.2–99.8)/ 100% (95.9–100.0)	100%/ 97.8% (91.8–99.4)	0.981 (0.954–1.000)

PPV: positive predictive value; NPV: negative predictive value; CI: confidence interval; sample type consisted of clinical specimens, dilutions of Accuplex controls, viral culture dilutions and assay controls.

**Table 2:** Precision overview of the FluoroType SARS-CoV-2 assay.

Viral Culture (Run in Triplicate)	*RdRP* Gene (Mean, SD, CV%)	*N* Gene (Mean, SD, CV%)
1:1000	30.0, 0.24, 0.96	32.2, 0.45, 1.4
1:10,000	33.5, 0.15, 0.46	35.1, 0.47, 1.35
1:100,000	36.7, 0.82, 2.23	38.9, 0.95, 2.45
1:1,000,000	38.75, 1.49, 3.83	-

**Table 3:** LoD of the FluoroType^®^ SARS-CoV-2 varID Q Ver 1.0 assay using dilutions of the Accuplex reference material.

Dilutions of Accuplex Wild-Type Reference Material (Copies/mL)	FluoroType^®^ SARS-CoV-2 Plus Ver 1.0		
	*RdRP* Gene (Ct)	*N* Gene (Ct)	Overall Result
Accuplex 5000	34.5	35.2	Positive
Accuplex 1000	36.9	38.6	Positive
Accuplex 1000	36.6	36.9	Positive
Accuplex 1000	36.3	37.8	Positive
Accuplex 500	37.3	37.7	Positive
Accuplex 500	37.4	39.4	Positive
Accuplex 500	36.2	36.7	Positive
Accuplex 250	38.2	39.7	Positive
Accuplex 250	37.2	37.8	Positive
Accuplex 250	38.1	37.9	Positive
Accuplex 100	39.2	−1	Positive
Accuplex 100	38.5	40.9	Positive
Accuplex 100	39.2	40	Positive

**Table 4:** SARS-CoV-2 variants profiles for gene targets included in the FluoroType SARS-CoV-2 assay.

VOCs	del69-70	E484K	N501Y	D80A
Alpha				
Beta				
Gamma				
Delta				
Omicron				

The blue shaded areas under each mutation indicate the variants in which the mutations will be detected. A sample with E484K, N501Y and D80A mutations will indicate it is a Beta variant. The FluoroType^®^ SARS-CoV-2 varID Q Ver 1.0 assay is unable to detect Delta variants.

## Data Availability

All data generated and analysed during this study are included in this published article and its Supplementary Information files.

## Supplementary Materials

The following supporting information can be downloaded at: https://www.mdpi.com/article/10.3390/diagnostics16131951/s1, Table S1: Results obtained on the various Standard of Care platforms for the residual specimens used in the study as well as results obtained using the FluoroType® SARS-CoV-2 plus Ver 1.0 assay. Green highlight indicates negative results; Red highlight indicates positive results; Orange highlight indicates invalid results obtained with the FluoroType® SARS-CoV-2 plus Ver 1.0 assay.
